# Social Media Data Mining of Antitobacco Campaign Messages: Machine Learning Analysis of Facebook Posts

**DOI:** 10.2196/42863

**Published:** 2023-02-13

**Authors:** Shuo-Yu Lin, Xiaolu Cheng, Jun Zhang, Jaya Sindhu Yannam, Andrew J Barnes, J Randy Koch, Rashelle Hayes, Gilbert Gimm, Xiaoquan Zhao, Hemant Purohit, Hong Xue

**Affiliations:** 1 Department of Health Administration and Policy College of Public Health George Mason University Fairfax, VA United States; 2 School of Computer Science and Engineering Changshu Institute of Technology Suzhou Jiangsu Province China; 3 Department of Physics and Engineering College of Engineering and Science Slippery Rock University of Pennsylvania Slippery Rock, PA United States; 4 Department of Health Behavior and Policy School of Medicine Virginia Commonwealth University Richmond, VA United States; 5 Department of Psychology College of Humanities and Sciences Virginia Commonwealth University Richmond, VA United States; 6 Center for the Study of Tobacco Products Virginia Commonwealth University Richmond, VA United States; 7 Department of Psychiatry School of Medicine Virginia Commonwealth University Richmond, VA United States; 8 Department of Communication College of Humanities and Social Sciences George Mason University Fairfax, VA United States; 9 Department of Information Sciences and Technology College of Engineering and Computing George Mason University Fairfax, VA United States

**Keywords:** tobacco control, social media campaign, content analysis, natural language processing, topic modeling, social media, public health, tobacco, youth, Facebook, engagement, use, smoking

## Abstract

**Background:**

Social media platforms provide a valuable source of public health information, as one-third of US adults seek specific health information online. Many antitobacco campaigns have recognized such trends among youth and have shifted their advertising time and effort toward digital platforms. Timely evidence is needed to inform the adaptation of antitobacco campaigns to changing social media platforms.

**Objective:**

In this study, we conducted a content analysis of major antitobacco campaigns on Facebook using machine learning and natural language processing (NLP) methods, as well as a traditional approach, to investigate the factors that may influence effective antismoking information dissemination and user engagement.

**Methods:**

We collected 3515 posts and 28,125 associated comments from 7 large national and local antitobacco campaigns on Facebook between 2018 and 2021, including the Real Cost, Truth, CDC Tobacco Free (formally known as Tips from Former Smokers, where “CDC” refers to the Centers for Disease Control and Prevention), the Tobacco Prevention Toolkit, Behind the Haze VA, the Campaign for Tobacco-Free Kids, and Smoke Free US campaigns. NLP methods were used for content analysis, including parsimonious rule–based models for sentiment analysis and topic modeling. Logistic regression models were fitted to examine the relationship of antismoking message-framing strategies and viewer responses and engagement.

**Results:**

We found that large campaigns from government and nonprofit organizations had more user engagements compared to local and smaller campaigns. Facebook users were more likely to engage in negatively framed campaign posts. Negative posts tended to receive more negative comments (odds ratio [OR] 1.40, 95% CI 1.20-1.65). Positively framed posts generated more negative comments (OR 1.41, 95% CI 1.19-1.66) as well as positive comments (OR 1.29, 95% CI 1.13-1.48). Our content analysis and topic modeling uncovered that the most popular campaign posts tended to be informational (ie, providing new information), where the key phrases included talking about harmful chemicals (n=43, 43%) as well as the risk to pets (n=17, 17%).

**Conclusions:**

Facebook users tend to engage more in antitobacco educational campaigns that are framed negatively. The most popular campaign posts are those providing new information, with key phrases and topics discussing harmful chemicals and risks of secondhand smoke for pets. Educational campaign designers can use such insights to increase the reach of antismoking campaigns and promote behavioral changes.

## Introduction

### Background

The current smoking rate among adults in the United States has steadily decreased from 20.9% in 2005 to 12.5% in 2020 [1,2]. Between 2019 and 2020, the prevalence of e-cigarette use among adults fell from 4.5% to 3.7% [2]. This significant decline in tobacco use can be attributed to a variety of effective national tobacco control strategies, including public health education campaigns, warning labels, smoke-free laws, and tobacco taxes [3]. Although 1-time federal or state policy changes, such as new tobacco taxes or smoke-free laws, have had a long-term impact on the general population [3], public health education campaigns are another important tool for facilitating behavioral changes among smokers [4]. As new tobacco products (eg, e-cigarettes) and media platforms (eg, streaming services and social media platforms) have emerged, antitobacco media campaigns have become an important strategy for promoting antitobacco attitudes and reducing smoking/vaping [5-7]. However, the majority of empirical studies on the effectiveness of such campaigns have focused traditional media campaigns [6-12], with limited evaluation of social media platforms. Therefore, timely evidence is needed to inform the adaptation of antitobacco campaigns to changing social media platforms.

Social media platforms, such as Facebook, Twitter, and YouTube, provide a valuable source of public health information, as one-third of US adults seek specific health information online [13,14]. Recognizing the critical importance of maximizing the use of these channels, in 2012, the Centers for Disease Control and Prevention (CDC) published guidelines for developing a social media communication strategy [15]. The framework has been used by state agencies and local health departments, which post health communication–related topics on Facebook and elsewhere [16]. The dissemination of information about antitobacco campaigns on social media differs greatly from traditional television advertising in that users in the newer approach participate more actively, while the latter is more passive. Moreover, there is a lack of evidence on how to best use these digital platforms for public health campaigns.

State and local governments and various nonprofit organizations have launched several mass media antitobacco campaigns (ie, the Real Cost campaign from the Food and Drug Administration [FDA] since 2014 [3,17] and campaigns from the Truth Initiative [18]). Early evidence suggests that youth or young adults exposed to these campaigns have a higher likelihood of smoking cessation [7]. For example, findings from the Real Cost campaign showed that increased levels of exposure to campaign advertising are associated with a significant increase in the odds of reporting agreement with campaign-specific beliefs that smoking is harmful. Other studies evaluating the effectiveness of the Truth Initiative and state-sponsored media campaigns showed that greater exposure to media campaigns is associated with lower smoking participation among youth on the individual level [19]. Other evidence, however, showed that youth smoking behavior is not associated with the degree of exposure to live broadcasts from the Truth Initiative [20], which suggests that in more recent years, the majority of youth moved away from broadcast television and gravitated instead toward social networks platforms [20]. During the same period, many campaigns recognized such trends among youth and have shifted their advertising time and effort toward digital platforms [21].

Although increasingly popular digital media platforms, such as web-based advertising and social media, allow campaigns to reach a larger audience, 1 of the major challenges for public health practitioners is determining where to invest resources, given the diverse media landscape and overwhelming number of platforms. Without a practical evaluation strategy suitable for campaigns on social media, campaign sponsors’ directions and decisions are also sometimes determined based on opinions or anecdotes rather than concrete, real-world empirical evidence, which is more useful for developing strategies to achieve campaign objectives [22]. Various evaluation metrics have been proposed for social media campaigns. User engagement, defined as the number of visits to a website; ad click-through rates; and the number of shares, likes, and comments on a social media site are popular evaluation measures of proximal impact—behavioral intentions lead to changes in behavior-related beliefs, attitudes, and social expectations [22,23]. Changes in beliefs and expectations have been shown to lead to behavioral intentions and further cause behavioral changes [23-26]. As a result, tracking user engagement with antitobacco messages and content on social media platforms may be more effective as a way to influence people's attitudes and formulate behavioral intentions.

However, only a limited amount of research has evaluated antitobacco campaigns on social media platforms. The majority of studies have focused on single campaigns with small sample sizes (ranging from as small as a few hundred to thousands of observations) [27-29]. Furthermore, the majority of the studies have used traditional analytical methods, such as manually coded content analysis—a time-consuming process. The implementation of natural language processing (NLP) for unstructured text mining in the field is easing such constraints. The recent innovations in NLP, such as bidirectional encoder representations from transformers (BERT) [30], enable the analysis of big unstructured data in social media platforms, including tracking adverse drug events and COVID-19 cases on Twitter [31,32].

### Novelty and Contribution to the Field

In this study, we aimed to conduct a novel content analysis of major antitobacco campaigns on Facebook using machine learning and NLP methods, as well as a traditional approach, to investigate the factors that may influence effective antismoking information dissemination and user engagement. The study has 3 main contributions to the health care field. First, this is among the first large-scale text-mining studies focusing on both large and small antitobacco educational campaigns. The study design leads to better generalizability. Second, we analyzed the sentiments in both campaign posts and comments, which provides valuable insight into the polarized association between posts and comments that can be used in future campaigns. Third, to be more specific in how the polarized association is presented, we conducted 2 types of content analysis: traditional manually coded and text labeled by topic modeling. This approach enabled us to observe specific topics and themes, in addition to the sentiments, that social media users are more interested in. The concept can be applied in future social media research.

## Methods

### Data Collection and Processes

We collected posts and comments from 7 large national and local antitobacco campaigns on Facebook between 2018 and 2021, including the Real Cost, Truth, CDC Tobacco Free (formally known as Tips from Former Smokers), the Tobacco Prevention Toolkit, Behind the Haze VA, the Campaign for Tobacco-Free Kids, and Smoke Free US campaigns (links to these campaign sites can be found in Multimedia Appendix 1, Table S3). We used Facebook Scraper [33] as well as manual collection to compile 3515 posts and 28,125 associated comments (see Multimedia Appendix 1, Tables S1 and S2, for concrete sample posts and comments). Not only did we collect text and emojis from these posts and comments, but we also collected information regarding the date/time of when the posts and comments were created, as well as whether the posts contained a video.

We then constructed sentiment scores—a quantitative measure that can detect polarity within the text, including the attitude, sentiments, evaluations, and emotions of the writer—for each post and its comments using VADER (Valence Aware Dictionary and Sentiment Reasoner) algorithms. VADER is a lexicon and rule-based sentiment analysis tool that is specifically attuned to sentiments expressed in social media [34]. The score is computed by summing the valence scores of each word in the lexicon, adjusted according to the grammatical and syntactical conventions that humans use when expressing or emphasizing sentiment intensity, and then normalized to be between –1 (most extreme negative) and +1 (most extreme positive). Following the literature, we set standardized thresholds to classify sentences as either positive (normalized score≥0.05), neutral (–0.05<normalized score<0.05), or negative (normalized score≤–0.05) [34]. The construct of the negative or positive sentiment for a post or comment is based on its text, and the context and relationship between posts and their comments do not affect the sentiment score. For example, if the original post is negative, a comment that is supportive of the original post can be either positive or negative depending on the texts of the comment. Here, we show 1 sample from the Real Cost campaign:

Negative post (sentiment score=–1): The movie poster is fake, but here’s a fact: The chemicals in cigarette smoke reach your lungs quickly every time you inhale. Your blood then carries the toxic chemicals to every organ in your body. #FakeMoviePoster #RealHorror #TheRealCost

The selected positive and negative comments are shown here:

Negative comments (sentiment score=–1): 
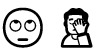

Positive comments (sentiment score=1): Keep up the good fight

More selected concrete sample posts and associated comments are provided in Multimedia Appendix 1, Tables S1 and S2). The agreement rate reached 77% between the machine learning algorithm and data manually checked from a randomly chosen set of 200 posts and comments. In this study, we tailored 3 methods for 2 different aims.

#### Statistical Modeling

In aim 1, we evaluated the effect of the framing strategy (ie, deemed positive or negative based on the sentiment score) of antitobacco campaign posts on the sentiment of users who engaged in them. The outcomes were (1) the sentiments of comments and (2) a binary indicator of whether a post received a higher-than-median number of likes, shares, and comments. The median was chosen as the threshold to relate our construct of sentiment scores [35]. The key exposures were the sentiments of posts. Logistic regressions were estimated and controlled for the number of likes, shares, comments, and monthly and yearly fixed effects. We clustered our estimates by posts in given campaign sites in our regression models to address the issue of intercorrelation between posts and comments from the same campaign sites. In total, 5 logistic regressions were fitted, and the detailed model specification was as follows:

Logistic regression 1:

Logit(у| positive or neutral comments)_ig_ = α + βx_ig_ + γz_ig_ + c + δ + u_ig_, i=1,…, n; g=1,…, G

The first logistic regression compared the comments that were deemed positive with those that were neutral. Here, subscript i denotes the unit of observation (individual comments) in post g; G denotes the number of posts; y denotes the sentiment of comments; and x is the indicator for the sentiment of posts, where 0 is neutrally framed posts, –1 is negatively framed posts, and 1 is positively framed posts. In addition, α is a constant term, z is a binary variable indicating whether the post g contains a video, c is the year fixed effect, δ is the monthly fixed effect, and u is an error term capturing unobservable variations.

Logistic regression 2:

Logit(у| negative or neutral comments)_ig_ = c + θx_ig_ + ∂z_ig_ + α + δ + u_ig_, i=1,…, n; g=1,…, G

The second logistic regression compared the comments that were deemed negative with those that were deemed neutral. Here, subscript i denotes the unit of observation (individual comments) in post g; G denotes the number of posts; y denotes the sentiment of comments; and x is the indicator for the sentiment of posts, where 0 is neutrally framed posts, 1 is negatively framed posts, and 2 is positively framed posts. In addition, c is a constant term, z is a binary variable indicating whether the post g contains a video, α is the year fixed effect, 𝛿 is the monthly fixed effect, and u is an error term capturing unobservable variations.

Logistic regressions 3-5:

Logit(у)_ig_ = d + τx_ig_ + ωz_ig_ + α + δ + h + u_ig_, i=1,…, n; g=1,…, G

The third to fifth logistic regressions were fitted to compare posts with and without more than the median number of likes, shares, and comments. Here, subscript i denotes the unit of observation (individual comments) in post g; G denotes the number of posts; y denotes the median number of likes, shares, or comments; and x is the indicator for the sentiment of posts, where 0 is neutrally framed posts, 1 is negatively framed posts, and 2 is positively framed posts. In addition, d is a constant term, z is a binary variable indicating whether the post g contains a video, α is the year fixed effect, δ is the monthly fixed effect, h is the hourly fixed effect, and u is an error term capturing unobservable variations.

#### Content Analysis and Topic Modeling

In aim 2, we looked into the details of the most commonly outlined content and topics formulated in the posts. We selected the top 100 liked and shared posts, as well as the top 100 posts with the most comments, to perform content analysis and topic modeling. For the content analysis, based on the literature, the content was categorized into 11 themes listed in Table 1 [36,37]. Two coders manually coded the theme for each post separately with an agreement rate of 87%.

**Table 1:** Themes in the content analysis.

Theme	Definition
Fear	Advertisements that aim to frighten
Humor	Advertisements that feature a humorous situation or dialogue
Sadness	Advertisements that present an emotionally unhappy scene to elicit heartache or anguish
Informational	Advertisements that present new information
Anger	Advertisements that provoke harsh, negative feelings
Perceived benefits	Advertisements that provide general information or guidelines about the benefits of quitting smoking/not starting smoking (eg, feel better, better health, and save money)
Perceived risks	Advertisements that provide general information or guidelines about barriers or disadvantages to quitting smoking/not starting smoking (eg, time constraint)
Perceived risks	Advertisements that provide general information or guidelines about the risks of smoking (eg, smoking causes cancer)
Self‐efficacy	Advertisements that mention the concept of self‐efficacy or its importance (eg, confidence building) in starting and maintaining smoking cessation/smoking prevention (eg, “You can do it.”)
Self‐affirmation	Advertisements that provide information regarding a recursive, self-perpetuation process that could motivate smoking cessation or smoking prevention and assess the user's personal self‐talk techniques (eg, “Do you tell yourself to quit smoking?”)
Subjective norm	Advertisements that provide general information or guidelines regarding how much significant others approve of smoking behaviors (eg, “Quitting smoking is a socially acceptable and encouraged activity; your spouse will love it if you quit smoking.”)

In addition to the traditional content analysis, we also used NLP methods for topic modeling (latent Dirichlet allocation [LDA]) [38]. LDA groups similar patterns (antismoking topics/word clusters/phrases in this study) from the collection of media texts into topic clusters. A more detailed schematic of the LDA algorithm can be found in Multimedia Appendix 1, Figure S1. This method has been shown in tobacco research to being a supplement for uncovering newly emerging topics that are not yet researched [39]. The Python packages the natural language toolkit (NLTK) [40], spaCy [41], and Gensim [41] were used. First, we tokenized the text of posts, converting uppercase letters to lowercase letters and removing stop-words and punctuation. Next, we used all the collected data to create a dictionary, using the same aforementioned top 100 shared, liked, and commented posts as tests of the results. We tested the optimal parameters for topic modeling to extract meaningful clusters [42] and identified that 5 topics with 4 words/tokens embedded in each were most representative and mutually exclusive.

## Results

### Changes in Posts, Comments, Likes, and Shares by Campaign

Table 2 shows the characteristics of response across 7 antitobacco campaign sites from 2018 to 2021. Between 2018 and 2021, 28,629 comments and 3746 of posts were collected. The level of engagement varied by campaign and year. The Real Cost campaign (the official campaign of the FDA) was highly active in 2018 but decreased drastically from 2019 to 2021. In 2018, there were 5728 total posts and comments, with an average of 4211 (SD 4134) likes, 272 (SD 197) comments, and 610 (SD 769.2) shares for each post. However, in 2019, the average likes (mean 23, SD 19), comments (mean 20, SD 26), and shares (mean 12, SD 12) shrank, and the decreasing trend remained in the next 2 consecutive years. The Truth Initiative, being 1 of the nation’s largest tobacco control organizations, experienced the highest user engagement over the 4-year study period, ranging from 800 to 9652 posts and comments. The other popular campaign site, Campaign for Tobacco-Free Kids, showed a different figure compared to the Real Cost and Truth Initiative campaigns. The user engagement was highest in 2021 with an average of 721 (SD 820) comments and an average of 4355 (SD 4011) likes to each post and 523 (SD 500) shares.

**Table 2:** Sample characteristics of posts and comments by year.

Characteristics		Overall	Behind the Haze VA	Campaign for Tobacco-Free Kids	Smoke Free US	The Real Cost	Tobacco Prevention Toolkit	Truth Initiative	﻿CDC^a^ Tobacco Free
**Overall**									
	Posts and comments, n (%)	28,629/28,629 (100)	130 (0.5)	6363 (22.2)	366 (1.3)	6140 (21.4)	421 (1.5)	13,997 (48.9)	1212 (4.2)
	Comments, mean (SD)	242 (422)	10 (14)	525 (758)	9 (14)	255 (201)	1 (3)	145 (174)	6 (9)
	Shares, mean (SD)	243 (472)	4 (8)	396 (471)	2 (2)	570 (758)	4 (6)	66 (73)	18 (21)
	Likes, mean (SD)	1711 (3127)	25 (45)	3150 (3882)	9 (6)	3930 (4128)	8 (7)	342 (507)	25 (27)
**2018**									
	Posts and comments, n (%)	7700/28,629 (26.9)	N/A^b^	N/A	N/A	5728/7700 (74.4)	3/7700 (0.04)	1969/7700 (25.6)	N/A
	Comments, mean (SD)	216 (197)	N/A	N/A	N/A	272 (197)	11 (0)	55 (57)	N/A
	Shares, mean (SD)	466 (708)	N/A	N/A	N/A	610 (769)	14 (0)	47 (42)	N/A
	Likes, mean (SD)	3199 (3982)	N/A	N/A	N/A	4211 (4134)	32 (0)	259 (811)	N/A
**2019**									
	Posts and comments, n (%)	11,537/28,629 (40.3)	60/11,537 (0.5)	1097/11,537 (9.5)	N/A	199/11,537 (1.7)	119/11,537 (1.0)	9652/11,537 (83.7)	410/11,537 (3.6)
	Comments, mean (SD)	157 (184)	16 (18)	24 (19)	N/A	20 (26)	1 (3)	184 (189)	10 (11)
	Shares, mean (SD)	78 (86)	8 (10)	88 (134)	N/A	12 (11)	4 (6)	82 (80)	28 (31)
	Likes, mean (SD)	351 (417)	20 (21)	116 (94)	N/A	23 (19)	10 (8)	403 (435)	38 (40)
**2020**									
	Posts and comments, n (%)	3208/28,629 (11.2)	56/3208 (1.7)	733/3208 (22.8)	179/3208 (5.6)	127/3208 (4.0)	228/3208 (7.1)	1576/3208 (49.1)	309/3208 (9.6)
	Comments, mean (SD)	37 (59)	6 (8)	62 (83)	11 (16)	11 (8)	1 (3)	44 (55)	4 (6)
	Shares, mean (SD)	27 (47)	1 (2)	67 (81)	2 (2)	12 (10)	5 (7)	19 (21)	14 (10)
	Likes, mean (SD)	94 (180)	36 (63)	234 (301)	8 (5)	18 (14)	9 (6)	74 (104)	19 (12)
**2021**									
	Posts and comments, n (%)	6184/28,629 (21.6)	14/6184 (0.2)	4533/6184 (73.3)	187/6184 (3.0)	86/6184 (1.4)	71/6184 (1.1)	800/6184 (12.9)	493/6184 (8.0)
	Comments, mean (SD)	541 (765)	0 (0)	721 (820)	8 (11)	6 (9)	0 (0)	90 (146)	4 (8)
	Shares, mean (SD)	387 (484)	0 (0)	523 (500)	2 (2)	5 (3)	1 (2)	17 (19)	12 (9)
	Likes, mean (SD)	3236 (3909)	1 (3)	4355 (4011)	11 (7)	8 (4)	3 (2)	327 (617)	17 (12)

^a^CDC: Centers for Disease Control and Prevention.

^b^N/A: not applicable.

### Associations of Positive vs Negative Framing Strategy with Comment Sentiment

Table 3 shows the effect of the framing strategy of Facebook posts on the sentiments of comments. Compared to neutral posts, positively framed posts generated more positive comments (odds ratio [OR] 1.29, 95% CI 1.13-1.48) as well as negative comments (OR 1.41, 95% CI 1.19-1.66). However, negatively framed posts were more likely to receive negative comments than neutral comments (OR 1.40, 95% CI 1.20-1.65) but not more positive comments. Of note is that the likelihood for negatively framed posts receiving negative comments did not differ from positively framed posts receiving positive comments (OR 1.40 vs OR 1.29, respectively; *F*_1_-score=0.98, *P*=.32).

**Table 3:** Effect of the framing strategy of Facebook posts on the sentiments of comments.

Framing of posts	Positive comments^a^	Negative comments^a^
Negative, aOR^b^ (95% CI)^c^	1.12 (0.98-1.28)	1.40^d^ (1.20-1.65)
Positive, aOR (95% CI)	1.29^d^ (1.13-1.48)	1.41^d^ (1.19-1.66)
Observations, N	19,838	19,670

^a^The reference group of the outcome variable was neutral comments.

^b^aOR: adjusted odds ratio.

^c^Estimates from logistic regressions with the reference group being neutral comments in the outcome variable were clustered by post ID. All the regressions were further controlled for the number of likes, shares, comments, monthly, and yearly fixed effects. Robust 95% CI values were clustered by post ID.

^d^*P*<.01.

### Associations of Positive vs Negative Framing Strategy with User Engagement

Table 4 depicts the effect of the framing strategy of Facebook posts on user engagement. Although the posts’ framing strategy did not relate to the number of likes, compared to neutral-framed posts, both negative (OR 2.42, 95% CI 1.43-4.09) and positive (OR 1.99, 95% CI 1.18-3.35) posts were more likely to have a higher-than-median number (62) of shares. We also noticed that posts containing a video were more likely to have more shares (OR 4.36, 95% CI 1.76-10.79). Similar findings were seen with regard to the number of comments.

**Table 4:** Effect of the framing strategy of Facebook posts on user engagement.

Framing strategy	More than the median number of likes (median N=294)	More than the median number of shares (median N=62)	More than the median number of comments (median N=104)
Negative framing^a^, aOR^b^ (95% CI)^c^	1.86 (0.99-3.50)	2.42^d^ (1.43-4.09)	2.42^d^ (1.37-4.26)
Positive framing^a^, aOR (95% CI)	1.76 (0.94-3.31)	1.99^d^ (1.18-3.35)	1.96^e^ (1.12-3.43)
Posts containing a video, aOR (95% CI)	1.69 (0.73-3.91)	4.36^d^ (1.76-10.79)	4.42^d^ (1.83-10.65)
Observations, N	28,629	28,629	28,629

^a^The reference group of the outcome variable was neutral comments.

^b^aOR: adjusted odds ratio.

^c^Estimates from logistic regressions were clustered by post ID. All regressions were further controlled for hourly trend, monthly fixed effects, and yearly fixed effects. Robust 95% CI values were clustered by post ID.

^d^*P*<.01.

^e^*P*<.05.

### Content Analysis of the Top 100 Ranked Posts

Table 5 presents the content analysis and topic modeling of themes observed from the top 100 ranked posts. Of the top 100 most liked, shared, and commented posts, informational (advertisements that present new information) posts (n=31, 31%) played a prominent role, followed by perceived risk (n=13, 13%) and self-affirmation (n=13, 13%) posts, which provided general information or guidelines about the benefits of quitting smoking or not starting smoking or own opinion related to the effects of tobacco usage on society. Fear (n=9, 9%), subjective norm (n=4, 4%), and humor (n=2, 2%) were less used strategies among these posts. Topic modeling provided a more precise image about key phrases or words used in the themes. For instance, we found a high percentage of posts that explained harmful chemicals (n=43, 43%) and different adverse outcomes related to the use of tobacco. Interestingly, the risk to pets (n=17, 17%), which explains the side effects of inhaling secondhand tobacco smoke on pets, attracted high user engagement.

**Table 5:** Content analysis and topic modeling of themes observed from the top 100 ranked posts.

Theme type and ranking^a^		Content analysis			Topic modeling	
		General theme	Posts, n (%)	Specific topics		Posts, n (%)
**Most liked**						
	1	Informational^b^	26 (26)	Contain harmful chemicals		21 (21)
	2	Perceived risks^c^	16 (16)	Cigarettes are risky		14 (14)
	3	Self-affirmation^d^	10 (10)	Cigarettes (e-cigarette) in relation to kids/youth		10 (10)
	4	Perceived benefits^e^	2 (2)	Risk to pets		6 (6)
	5	Fear^f^	2 (2)	Flavored Juul (e-cigarettes)		3 (3)
**Most shared**						
	1	Informational	29 (29)	Smoke leading to adverse outcomes		23 (23)
	2	Perceived risks	17 (17)	Contain harmful chemicals		12 (12)
	3	Self-affirmation	12 (12)	Cigarette (flavored) use in youth is an epidemic		6 (6)
	4	Subjective norm^g^	4 (4)	Flavored menthol (e-cigarettes)		5 (5)
	5	Fear	4 (4)	Risk to pets		5 (5)
**Most commented**						
	1	Informational	38 (38)	Smoking and health		15 (15)
	2	Self-affirmation	17 (17)	Contain harmful chemicals		10 (10)
	3	Perceived risks	6 (6)	Cigarette (flavored) use in youth is an epidemic		8 (8)
	4	Fear	3 (3)	Risk to pets		6 (6)
	5	Humor^h^	2 (2)	Flavored menthol (e-cigarettes)		6 (6)

^a^Here, the ranking was presented from 1 to 5, and the numbers and percentages for each category did not sum up to 100. In addition, there might be overlaps among the most liked, most shared, and most commented posts. However, this did not affect the interpretation of the results.

^b^Informational: advertisements providing information to the society about the latest news related to tobacco.

^c^Perceived risks: advertisements providing information about risks associated with the use of tobacco.

^d^Self-affirmation: advertisements providing general information or guidelines operating through recursive, self-perpetuating processes, motivating individuals to capitalize on pre-existing resources to facilitate change.

^e^Perceived benefits: advertisements providing information about advantages after quitting tobacco.

^f^Fear: advertisements that aim to frighten.

^g^Subjective norm: advertisements providing general information or guidelines for the perception of how much significant others approve of smoking behaviors.

^h^Humor: advertisements that feature a humorous situation or dialogue.

## Discussion

### Principal Findings

In this novel study, we deployed NLP, traditional content analysis, sentiment analysis, and regression analysis to assess factors that influence effective antismoking information dissemination and user engagement. We found that large campaigns from government and nonprofit organizations have more user engagement compared to local and smaller campaigns. Although positive posts tend to receive more positive comments, Facebook (now named META) users, in general, are more responsive to negative posts, leaving more comments (both negative and positive). Our content analysis and topic modeling uncovered that most popular campaign posts tend to be informational (ie, providing new information), where the key phrases include talking about harmful chemicals (43%) as well as the risk to pets (17%).

Large campaigns of government and nonprofit organizations (ie, the Real Cost from the FDA and the Truth Initiative) on Facebook are active, in which campaign posts on average receive more comments, shares, and likes. These flagship programs have dedicated numerous resources to design, promote, and reach their target populations [3,7,19,43]. Traditionally, the program evaluation of antitobacco campaigns on mass media has relied on reach and frequency, and the combination of the two, to measure exposure. However, this approach has significant weakness when applied to digital media platforms compared to traditional media (eg, television ads) [22]. Because each social media platform has its own characteristics, it is difficult to have a standardized measurement. In social media research, likes, shares, and comments can serve as a proxy measurement of user engagement [22]. However, using this click-through indicator sometimes leads to a confounded result. For instance, evidence shows that for any individual antismoking message, Facebook has the highest as well as the lowest click-through rates when compared with Twitter and Instagram. In other words, when estimating message engagement using the click-through rate, the estimates from Facebook would have been averaged out due to its extreme nature and hence biased toward 0 [29]. Although we demonstrated that large and well-designed campaigns are more highly engaged in by users [43], the drastic variations demonstrated the difficulty in accurately measuring user engagement on social media platforms [27].

To better measure user engagement, we constructed sentiment scores and performed sentiment analysis. Our findings revealed that Facebook users, in general, are more responsive to negative posts by leaving more comments (both negative and positive). This provides an important insight for program designers. Previous evidence in experimental psychology research shows that only in the case of a negative message do participants consider a news-labeled message more important than a rumor-labeled or a nonlabeled message [44], a well-known behavior called negativity bias [45,46]. Recent analysis from Twitter also indicated the existence of negativity bias on social media, where researchers found that negative campaign advertising is more likely to mobilize incivility [47]. The fact that ordinary internet and social media users are more responsive to negative messages than positive ones provides an important insight for future program design and message dissemination. Education campaign designers can use such psychological patterns (ie, negativity bias) to increase user engagement and potentially broaden the base of their audience.

To obtain more insight into topics and themes that social media users are more interested in, we conducted content analysis and novel machine learning analysis of topics. Our work showed that the top 100 popular campaign posts tended to be informational (ie, providing new information), where the key phrases/words used included but were not limited to talking about harmful chemicals (43%) and risk to pets (17%). The findings echo previous work on sentiment analysis, which showed that people are more responsive to negative messages and especially information related to overlooked risks. It is intriguing and especially important to observe that social media users pay attention to and engage in posts related to risks involving their pets. About 56.8%-67.0% of Americans have pets (mostly dogs and cats) in their households [48], and many of them consider pets as family members. Previous evidence indicates that 1 of the major reasons smokers quit smoking is to improve the health of their family members [49]. Although traditional academic research defines official family members as husband/wife and children, our results shed light on the fact that pets can be important parts of a household and, thus, a basis for future educational programs to design messaging that is more flexible and relevant to people’s lives. More specifically, messages that are unexpected and counterintuitive but meaningful to their daily life (eg, focusing on all those who might be important to them) could better generate behavioral changes among smokers.

### Strengths and Limitations

Although we used a novel approach with a large sample size to determine the factors that may influence effective antismoking information dissemination and user engagement, several limitations are worth noting in this study. First, the data structure was cross sectional, and we were not able to track individuals’ behavior changes (ie, we only observed the comments left by a person but were unable to observe whether they actually cut down, quit, or decided not to start smoking afterward). Measuring the intention to quit on social media, however, serves as a significant goal for future longitudinal studies. Second, we only selected some of the largest and most active educational campaigns, focusing on 1 social media site. Our estimates might not be able to generalize to all antitobacco campaigns and all populations. Third, although the algorithm used for sentiment analysis can decipher many emojis, it is unable to distinguish sarcasm presented in texts or emojis, so readers should be aware and interpret the results with caution, since the estimates could be biased. Fourth, there could be an intercorrelation between posts and comments from the same campaign sites. To address this issue, we specifically clustered our estimates by posts in given campaign sites in our regression models.

### Conclusion

Facebook users tend to engage in antitobacco educational campaigns that are framed negatively. The most popular campaign posts are those providing new information, with key phrases and topics discussing harmful chemicals and risks of secondhand smoke for pets. Educational campaign designers can use such insights to increase the reach of antismoking campaigns and promote behavioral changes.

Future research could focus more on 3 specific areas. First, this study was cross sectional. To obtain better causal estimators, a longitudinal design that tracks unique user IDs, seeing how their reaction evolves and whether those reactions can turn into the action of quitting, is desirable. Second, the sample collection in future studies can combine multiple platforms (ie, Twitter, Instagram, YouTube) to obtain a larger data quantity. In addition, a comparison among different platforms can be made to evaluate differences in the user response, which future campaigns can use to nudge the target population. Third, we acknowledge that the current state-of-the-art NLP is BERT. Being a contextual NLP model, BERT could be more effectively used to discrete sarcasm—1 limitation of this study—and generate sentiment scores and topics with improvements in accuracy.

## Appendix

Multimedia Appendix 1 Supplementary tables and figure.

## Abbreviations

BERT: bidirectional encoder representations from transformers
CDC: Centers for Disease Control and Prevention
FDA: Food and Drug Administration
NLP: natural language processing
OR: odds ratio
