# Supplementary material for: Social Media Data Mining of Antitobacco Campaign Messages: Machine Learning Analysis of Facebook Posts
Source: J Med Internet Res. 2023 Feb 13;25:e42863. doi: 10.2196/42863 (PMC9972210; doi:10.2196/42863)
Supplement: Multimedia Appendix 1 [file jmir_v25i1e42863_app1.docx]

# Appendix

eTable1. Selected sample comments

eTable2. Selected sample posts

eTable3. Links to the anti-tobacco campaign sites

eFigure1 Schematic of LDA model

eTable1. Selected sample comments

| Post id | | Comment text | | Sentiment Score |
| --- | --- | --- | --- | --- |
| 5496 | | Ban Newports. | | -1 |
| 5496 | | Keep up the good fight | | 1 |
| 5496 | | Great cause. |  | 1 |
| 5496 | | 🙄🤦🏼‍♂️ |  | -1 |
| 5496 | | Banning flavored vapes only pushes sales to New Hampshire and the black market .. where kids are buying this illegally online. NOT in state approved stores that ID | | -1 |
|  |  | |  |  |
| 5498 | People really on here being pro lung disease | | | 1 |
| 5498 | Am I allowed to hold one while smoking a cigarette? | | | 1 |
| 5498 | How does it feel to be a sellout? | | | -1 |
| 5498 | “1 JuUl pOD = 20 cIGarETtEs WorTh oF NIcoTiNE”... uhhhh that’s kinda the point. 🤦🏼‍♂️ | | | 0 |
| 5498 | And people still smoking 😂 | | | 1 |

eTable 2. Selected sample posts

| **Post id** | **text** | **Score** |
| --- | --- | --- |
| **25238** | The movie poster is fake, but here’s a fact: The chemicals in cigarette smoke reach your lungs quickly every time you inhale. Your blood then carries the toxic chemicals to every organ in your body. #FakeMoviePoster #RealHorror #TheRealCost | -1 |
| **15995** | Despite JUUL Labs' previous claims that it only marketed to adult smokers, a new study we co-authored found that almost half of individual users following the @JUULvapor Twitter account are underage youth.  REUTERS.COM Teens made up most of e-cigarette maker Juul's Twitter following -... | 0 |
| **19675** | We’ve spent years fighting to bring the tobacco epidemic out of the shadows. Now there’s a new crisis that also needs our attention. Thanks to NBC News for helping us amplify the launch of our opioid education and prevention campaign. More: http://bit.ly/truthopioids  NBCNEWS.COM Graphic new ads drive home the desperation fueling the opioid crisis | -1 |

Notes: Data in some campaign sites were not completely collected. For instance, we were only able to collect posts and comments from CDC Tobacco Free between 2020 and 2021. In addition, not all comments are collected. Some posts with comments were not available by the collecting algorithm due to restrictions imposed by Facebook.

eTable 3. Links to the anti-tobacco campaign sites

| Campaigns | Link |
| --- | --- |
| The Real Cost | https://www.facebook.com/KnowTheRealCost |
| Truth | https://www.facebook.com/truthorange |
| CDC Tobacco Free (formally known as Tips from Former Smokers) | https://www.facebook.com/cdctobaccofree |
| Tobacco Prevention Toolkit | https://www.facebook.com/TobaccoPrevToolkit |
| Behind the Haze VA | https://www.facebook.com/BehindTheHazeVA |
| Campaign for Tobacco-Free Kids | https://www.facebook.com/tobaccofreekids |
| Smoke Free US | https://www.facebook.com/SmokefreeUS |

eFigure1 Schematic of LDA model , modified from Buenaño-Fernandez et al.,^1^


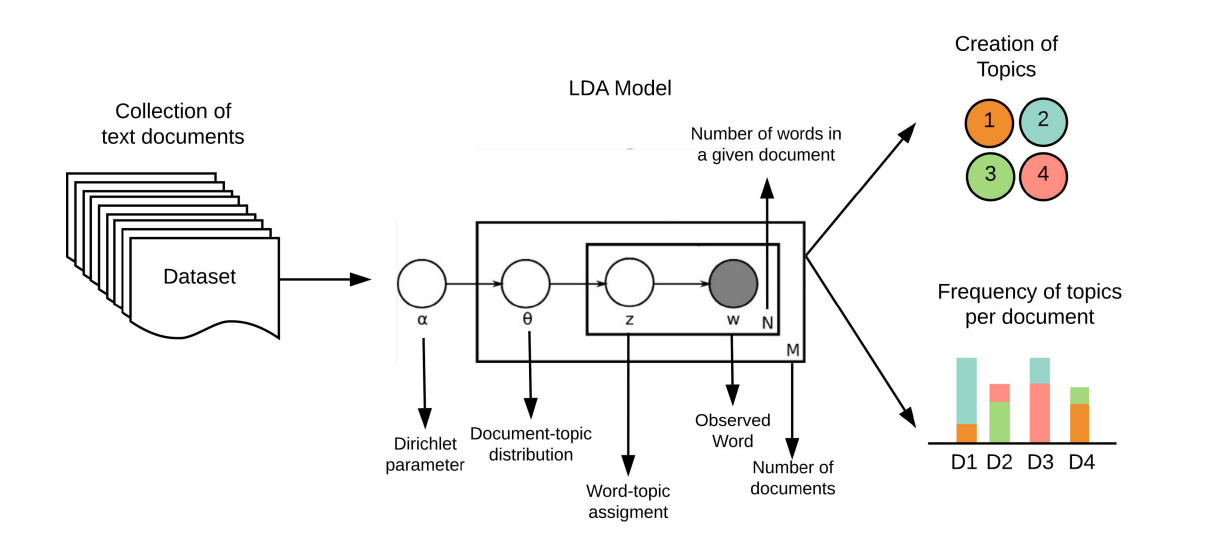


Reference

1. D. Buenaño-Fernandez, M. González, D. Gil and S. Luján-Mora, "Text Mining of Open-Ended Questions in Self-Assessment of University Teachers: An LDA Topic Modeling Approach," in *IEEE Access*, vol. 8, pp. 35318-35330, 2020, doi: 10.1109/ACCESS.2020.2974983.
